# Assessing the barriers and facilitators of climate action planning in local governments: a two-round survey of expert opinion

**DOI:** 10.1186/s12889-023-16853-8

**Published:** 2023-10-05

**Authors:** Steven Dodd, Scott Butterfield, Jessica Davies, Mette Kragh Furbo, Abigail Morris, Heather Brown

**Affiliations:** 1 Division of Health Research, Lancaster University, Lancaster, UK; 2 Blackpool Council, Blackpool, UK; 3 Lancaster Environment Centre, Lancaster University, Lancaster, UK

**Keywords:** Climate change, Barriers and facilitators, Health inequalities, England, Local government

## Abstract

**Background:**

Climate change is one of the greatest threats to public health in this century. The UK is one of six countries that has enshrined in law a commitment to become net zero by 2050. However, there is a lack of guidance and structure for local government in the UK, which has responsibility for public health, to reach this goal and help their communities mitigate and adapt to the health and health inequality impacts of climate change. This study aimed to identify common barriers and facilitators related to addressing the health and health inequality impacts of climate change in local governments.

**Methods:**

Using Normalisation Process Theory, we developed a two-round survey for people working in local authorities to identify the barriers and facilitators to including the health and health inequality impact of climate change in their climate action plans. The survey was delivered online via Qualtrics software. In the first-round respondents were able to express their views on barriers and facilitators and in the second round they ranked common themes identified from the first round. Two hundred and fifty people working in local government were invited to take part and *n* = 28 (11.2%) completed the first round of the survey and *n* = 14 completed the second round. Thematic analysis was used in Round 1 to identify common themes and weighted rankings were used to assess key barriers and facilitators in Round 2.

**Results:**

Key facilitators were the need to save money on energy, and successful partnership working already in place including across local government, with local communities and external stakeholders. Key barriers were insufficient staff, resources and lack of support from management/leaders, and lack of local evidence.

**Conclusion:**

To mitigate and adapt to the health impacts of climate change, local government must nurture a culture of innovation and collaboration to ensure that different departments work together This means not just working with external partners, but also collaborating and co-producing with communities to achieve health equity and mitigate the debilitating effect of climate change on public health.

**Supplementary Information:**

The online version contains supplementary material available at 10.1186/s12889-023-16853-8.

## Background

With its diverse and pernicious effects, climate change constitutes a looming and escalating risk to public health in the UK, with the potential to trigger a cascade of adverse impacts on infectious disease control, mental health, air pollution-related illnesses and heat-related morbidity and mortality [1, 2]. The poor and marginalised tend to shoulder the burden of climate injustice [3], with future changes likely to only further deepen existing health inequalities [4, 5]. In this concerning context, local authorities are ideally placed to act decisively to safeguard local communities and preserve public health from climate risk. By fulfilling their obligations to local populations, local authorities can bolster community resilience and contribute to the creation of a healthier and more cohesive society.

According to the UK’s Office of National Statistics, net zero means that 'the UK’s total greenhouse gas (GHG) emissions would be equal to or less than the emissions the UK removed from the environment. This can be achieved by a combination of emission reduction and emission removal such as carbon sequestration through reforestation or sustainable agriculture practices [6].

**Fig. 1:**
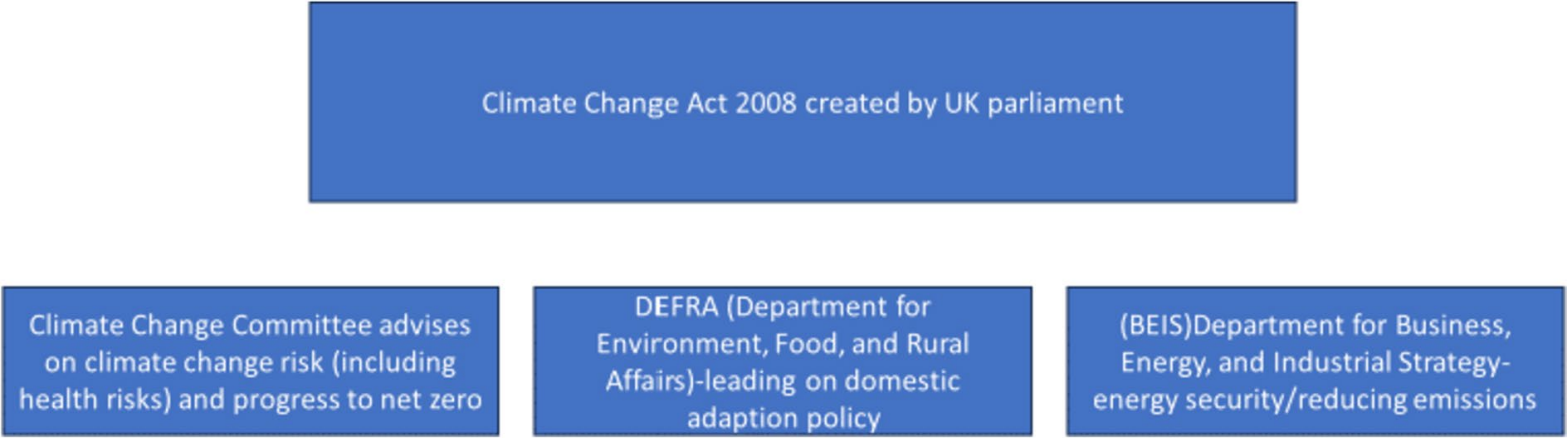
Statutory responsibility for climate change policy

An overview of statutory responsibilities for climate change policy as set out in the Climate Change Act 2008 are described in Fig. 1. The UK Climate Change Act 2008, commits the UK government to achieving net zero by 2050. To achieve this, there is the Climate Change Committee who advises on climate change risks including health risks and progress to net zero. The two main government departments for climate change are: Department for Environment, Food, and Rural Affairs (DEFRA) who advises on adaption and Department for Business, Energy, and Industrial Strategy (BEIS) on advises on emissions. There are separate climate change policies for the devolved governments of Northern Ireland, Wales, and Scotland. The legislative framework skews away from giving local authorities’ responsibility.

To achieve the UK’s net zero target by 2050 more than half of all cuts in emissions need to be made by people and businesses adopting low-carbon solutions. These decisions are made at a local rather than a national level and are reliant on having appropriate infrastructure and systems in place to facilitate a change in behaviour [7]. Local authorities have direct control of approximately 2–5% of emissions in their area. However, with their responsibilities for public health, housing, planning, economic development, education skills delivery and risks from events such as flooding, local authorities can foster partnerships between the local community, businesses and third sector organisations to support their local area’s health and economic well-being and play an important role in developing local solutions to climate change [8]. Despite this potential for local leadership and innovation, local authorities have been held back by inadequate powers, and lack of skills and capacity caused by a reduction in funding to local authorities from central government since 2010 [8]. Local government obligations for adaptation include flood risk management, preparation for emergencies, and the requirement to proactively adapt through planning decisions [9] but research suggests action could be better supported in a number of ways, including ‘improved public health intelligence, concise communications, targeted support, visible local and national leadership and clarity on economic costs and benefits of adaptation’ [10]. As can be seen in Fig. 1 above, nationally, there is no overall plan on how local authorities fit into the UK’s net zero plans.

Instead, local authorities independently tackle mitigation and adaptation by developing climate action plans, including indicators with which to chart their progress. There is no requirement that these plans consider the health and health inequality impact of climate change when the climate action plan is developed. The lack of any centralised coordination of local authority policies contributes to a risk that ambitious hopes for local government-led change on climate and health fail to be realised in practice [11].

The aim of our research was to identify common barriers and facilitators related to the health and health inequality impacts of climate change faced by local authorities as they attempt to implement their climate action plans. We conducted a two-round survey with climate change/public health officers employed in local government about barriers to, and facilitators of, creation of high quality-policies about the health and health inequality impacts of climate change that incorporate external expertise, local insights, and effective implementation strategies. To provide guidance to local authorities, we focused on areas through which local authorities can use their influence to facilitate the transition to net zero. These included 1) perceived barriers and facilitators towards collaborating/co-constructing health/health inequality-related components of climate action plans with local communities (partnership working); 2) barriers and facilitators to using external evidence to support the development of health/health inequality-related components of climate action plans; and 3) barriers or facilitators to implementation of the health/health inequality-related components of climate action plans.

This evidence can be used to support local authorities and their partners to help consider and address the health challenges posed by climate change. By identifying barriers, we can show where certain areas may need additional support to reduce the risk of additional inequalities arising. By identifying facilitators, we can provide suggestions of what may be useful to develop climate action plans that consider the health and health inequality impacts of climate change. As a whole, this piece of research contributes by providing evidence to help with the implementation of effective climate action plans that integrate health considerations. It fits into a small but growing literature on the topic of local government climate policy in the UK [11–18]. To the best of our knowledge, it is one of very few [16, 10] studies to address the barriers and facilitators to considering the health challenges of climate change and how local government can address these challenges.

## Methods

### Overview of the two-round survey process

Our approach was informed by Normalisation Process Theory (NPT) [19]. NPT examines the factors that promote or inhibit interventions becoming part of everyday practice. It seeks to explain not only how these interventions function in early implementation, but also when they are fully embedded into routine practice and are normalised. We chose to use NPT as a conceptual framework for this study because it provides a framework for understanding how new practices, such as considering the health and health inequality impacts of climate change in climate action plans, become normalized and embedded in routine practice. NPT involves four constructs that shape the normalisation process: coherence, cognitive participation, collective action and reflexive monitoring [20, 21]. Coherence refers to the process of sense making that people must go through as they become familiar with an intervention. Cognitive participation focuses on the commitment and engagement of people in the implementation process. Collective action is the work that people have to participate in to implement the new practices. Lastly, Reflexive monitoring is concerned with the ongoing appraisal and evaluation of the new practice and its impact by the people involved.

We developed and delivered a two-round survey to investigate the opinion of local authority climate change/public health officers on the barriers and facilitators they experience in creating and implementing policies to reduce the health risks of climate change and achieve potential co-benefits such as 'improvements in public health, reduced NHS costs, greater energy security, growth in the low-carbon jobs market and a reduction in poverty and inequality' [22]. To reduce the health risks this would include both adapting to climate change such as flood defences, air conditioning, well-insulated buildings as well as through mitigation measures to reduce the negative impacts of climate change and the associated health risks such as implementing measures to encourage the adoption and use of electric vehicles by transport companies and individuals which has benefits on air quality and carbon emissions; restoring nature in urban areas which is where mitigation and adaptation are linked – planting trees help to sequester carbon as well as providing canopy cover to reduce sun exposure’.

Our two-round approach enabled us to first allow respondents to express their views on the key issues and then to gauge how they ranked these issues in order of importance. We used an online form of the survey in which participants completed two rounds of questionnaires sent to them via the Qualtrics platform [23]. Before the survey began, a member of the team (MK) carried out an online search to identify publicly available contact information for local authority climate change officers (or equivalent role) or public health officers from the UK. We sent the survey to all people working in a local authority who worked in either the climate change or public health team We then sent a link to the survey, along with an overview of the study and the rationale for using the two-round survey.

We identified 250 local government officers who were approached to take part, (*n* = 28) completed the first round of the survey (11.2% response rate). Fifty percent of the 28 respondents (*n* = 14) took part in the second round.

### Round 1 (see Additional file 1: appendix 1)

The first-round questions included a number of open questions designed to invite responses that could inform the second round of the survey (see Additional file 1: appendix 2).

### Round 2 (see Additional file 1: appendix 2)

Participants who had consented to take part in a second-round questionnaire were sent a follow-up survey about one month after the first. This survey was based upon the themes which were identified from the Round 1 survey. Participants were asked to rank six lists of barriers and facilitators, and one list of overall priorities in order of importance. We decided to use rank ordering questioning so that we could understand the priority given to different barriers and facilitators in local authorities' climate action plans and to capture participant's relative judgments. Rank order questioning enables us to explore patterns and common themes across different questions, facilitating an exploration of the relative importance of different factors'.

### Analysis

Responses to Round 1 questions were analysed to create lists of barriers and facilitators that respondents were asked to rank in order of importance in the second round of the survey. To create the questions for the second round, responses from round one was thematically coded by SD using NVivo and grouped into themes that captured the overarching concepts with fewer categories.

Responses to round 2 were analysed by rank order analysis to find the themes that were most highly ranked as barriers and facilitators to action on the health/health inequality aspects of climate change. For a given question, responses were tabulated to show the rank order choices made by each participant for a given question. These choices were used to create a frequency table counting the number of times each option was awarded a given rank. For example, in the table below for question 4, 'collaboration with academic partners' was ranked in first place by two respondents, in second place by five respondents, and in third place by seven respondents Table 1.

**Table 1:** Example frequency table for round two responses

Rank	**Collaboration with academic partners**	**Commitments to adequately staffing and resourcing research capacity**	**Greater collaboration between climate and public health teams**
1	2	2	10
2	5	6	3
3	7	6	1

Rank shows the highest rated by number of respondents. The column shows the number of respondents who reported each of the column headings as most important

In Table 2, we show how the data from the frequency tables was used to generate a weighted total score for each option based on the rankings allocated to it. Data from question 4 is used in Table 2, for every rank of 1 (Ranked 1st of 3 options) the option was given a score of 3, for every ranking of 2 (2nd of 3 options) the option receives a score of 2, and for every ranking of 3 it receives a score of 1. In Table 2 we can see that using this methodology, this means the highest overall score is given to the option that was ranked highest by respondents.

**Table 2:** Example ranking table for round 2 responses

	**Total weighted score**	**Rank**
Greater collaboration between climate and public health teams	**37**	**1**
Commitments to adequately staffing and resourcing research capacity	**24**	**2**
Collaboration with academic partners	**23**	**3**

Total weighted score shows the number of respondents that reported the row heading as the most important factor which is multiplied by 3 if ranked as most important, 2 if ranked as second most important and 1 if ranked in 3. Rank is defined in Table 1

## Results

Table 3 presents participant characteristics. Although the sample was quite small, we achieved diversity among respondents, both in terms of geography and job role/seniority. The majority of respondents in round 1 and round 2 were Climate Change or Sustainability Officers, which is to be expected given the nature of role responsibilities and key contacts given in climate action plans.

**Table 3:** Participants characteristics

	Round 1	Round 2
Region of respondent’s local authority	South East: 32% (*n* = 9)	South East: 29% (*n* = 4)
	South West: 14% (*n* = 4)	South West: 21% (*n* = 3)
	East Midlands: 7% (*n* = 2)	East Midlands: 7% (*n* = 1)
	West Midlands: 7% (*n* = 2)	West Midlands: 14% (*n* = 2)
	North West: 32% (*n* = 9)	North West: 21% (*n* = 3)
	Wales: 4% (*n* = 1)	Undeclared: 7% (*n* = 1)
	Undeclared: 4% (*n* = 1)	
Profession	Climate Change/Sustainability Officer: 57% (n-16)	Climate Change/Sustainability Officer: 57% (*n* = 8)
	Climate Change/Sustainability Manager: 25% (*n* = 7)	Climate Change/Sustainability Manager: 29% (*n* = 4)
	Public Health Professional: 18% (*n* = 5)	Public Health Professional:14% (*n* = 2)
Time in current role		0 -1 years of service: 36% (*n* = 5)
	0–1 years of service: 43% (*n* = 12)	2–5 years of service: 57% (*n* = 8)
		6 + years of service: 7% (*n* = 1)

### Results from Round 1

Table 4 is a summary of the results from the round 1 survey that informed the questions in round 2. The first question asks about barriers that may have limited a council’s use of externally produced evidence in formulating and implementing health-related components of their Climate Action Plans. The most common challenge when using external evidence was finding the time and resources to assess its quality and relevance (*n* = 14). Some councils also lacked the expertise to do this (*n* = 4) or struggled to find evidence that matched their local context (*n* = 3). Other barriers included difficulty accessing the evidence (*n* = 2), lack of support from senior leaders (*n* = 1) and lack of local political backing (*n* = 1).

**Table 4:** Responses from Round 1 that informed questions in Round 2

Please summarise any barriers you believe may have limited your council's use of externally produced evidence in the formulation and implementation of the health-related components of its Climate Action Plan	Please describe what you think are the greatest barriers to working with local communities when developing the health-related components of a Climate Action Plan	Please summarise the barriers to implementing the health-related components of your council's Climate Action Plan	Please summarise the risks to public health included within the Climate Action Plan (priorities)
Competing demands/lack of resources and time required to assess the evidence (*n* = 14)	Lack of capacity/resources to engage with those outside of the council (*n* = 12)	Insufficient staff and resources (*n* = 9)	Flooding (*n* = 9)
Lack of expertise required to assess the evidence (*n* = 4)	Lack of interest/knowledge within the local community (*n* = 8)	Organisational culture, values/awareness of colleagues (including senior leadership (*n* = 3))	Air quality and associated health risks (*n* = 8)
Lack of evidence at a local level (*n* = 3)	Challenges reaching specific communities or demographics (*n* = 6)	Difficulties engaging with colleagues from the healthcare system (*n* = 3)	Heatwaves (*n* = 6)
Inaccessibility of the evidence (*n* = 2)	Community-council relations and lack of trust in the council (*n* = 4)	Too little is known about the health impacts of climate change (*n* = 3)	Improving the quality of the housing stock (*n* = 4)
Lack of commitment on the part of senior leadership (*n* = 1)	Lack of pre-existing links to the local community (*n* = 1)	The difficulty of understanding the health effects of climate change (*n* = 2)	Fuel poverty and energy use (*n* = 4)
Lack of political support at a local level (*n* = 1)	Colleague's unwillingness to work with local communities (*n* = 1)	Difficulties engaging with local communities and stakeholders (*n* = 2)	Unequal impacts (*n* = 3)
	Preference for working with other public sector organisations (*n* = 1)	Priority is narrowly on reducing emissions and not on health (*n* = 2)	
		Local political context not conducive (*n* = 2)	
		Council colleagues working in silos, lack of joining up and collaboration (*n* = 1)	
		National political and policy context not conducive (*n* = 1)	
		Health not thought to be at risk from climate change in your locality (*n* = 1)	

The second question asks about barriers to working with local communities when developing health-related components of a Climate Action Plan. The most common barrier reported was having enough capacity or resources to reach out to them (*n* = 12). Some communities were not thought to be interested or informed about climate change and health (*n* = 8) or were thought to be hard to engage (*n* = 6). Other barriers included poor relations or trust between communities and councils (*n* = 4), lack of existing links with communities (*n* = 1), reluctance of colleagues to involve communities (*n* = 1) and preference for working with other public sector organisations (*n* = 1).

The third question deals with barriers to implementing health-related components of council’s Climate Action Plans. The biggest barrier was not having enough staff and resources to carry out the plan (*n* = 9). Other barriers included organizational culture and values or awareness of colleagues including senior leadership (*n* = 3), difficulties engaging with colleagues from the healthcare system (*n* = 3), and too little being known about the health impacts of climate change (*n* = 3). Some also said they found it hard to understand the health impacts of climate change (*n* = 2) or had difficulties engaging with local communities and stakeholders (*n* = 2). Another challenge was how to focus on health in their plan. Some respondents said their council was mainly concerned with reducing emissions rather than improving health (*n* = 2). Others said they faced political barriers at a local (*n* = 2) or national level (*n* = 1) that prevented them from prioritising health. One respondent also said that health was not thought to be at risk from climate change in their area.

The fourth column summarizes risks to public health included within respondent's Climate Action Plans. The most commonly reported risk was flooding (*n* = 9) followed by air quality and associated health risks (*n* = 8), heatwaves (*n* = 6), improving the quality of housing stock (*n* = 4) fuel poverty and energy use(*n* = 4) and unequal impacts(*n* = 3).

### Results from Round 2

#### Question 1. The most significant barriers to implementation of health/health inequality-related components of local authorities' climate action plans

**Fig. 2:**
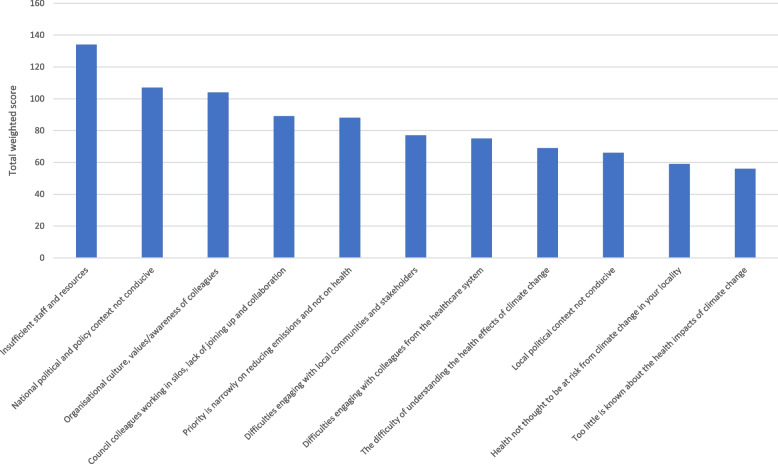
The most significant barriers to implementation of health/health inequality-related components of local authorities' climate action plans

In Fig. 2, the highest ranked barrier to implementation of health/health inequality-related components of climate action plans was insufficient staff and resources. Of the other barriers to implementation, we see that the national political and policy context was the second highest ranked barrier to action. The third highest ranked barrier concerned problems internal to the council such as organisational culture and values/awareness of colleagues (including senior leadership). Internal problems are also evident in the fourth ranked option of 'council colleagues working in silos, lack of joining up and collaboration'.

#### Question 2: the most significant facilitators of the implementation of health/health inequality-related components of local authorities' climate action plans

**Fig. 3:**
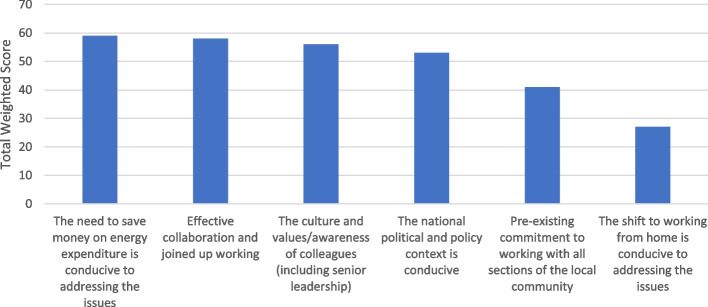
The Most Significant Facilitators of Implementation of Health/Health Inequality-related Components of Local Authorities' Climate Action Plans

In Fig. 3, first-ranked among facilitators of implementation was 'the need to save money on energy expenditure', serving as a financial incentive for more urgent action. Closely following behind in second place was 'effective collaboration and joined up working' and in third place 'the culture and values/awareness of colleagues'. Also receiving support in its ranking of fourth place as a facilitator was "the national political and policy context is conducive", suggesting some participants perceive there to be a degree of support from national government and policymakers.

#### Question 3: the most significant barriers to local authorities' use of external evidence to inform the health/health inequality-related components of climate action plans

**Fig. 4:**
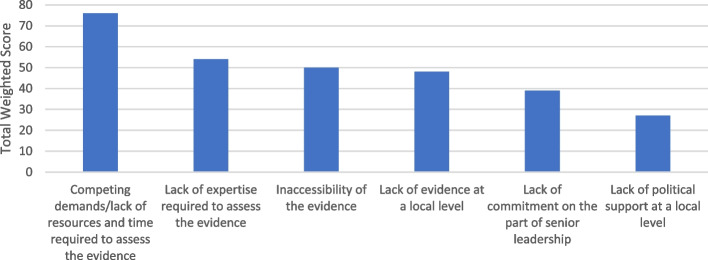
The most significant barriers to local authorities' use of external evidence to inform health/health inequality-related components of climate action plans

In Fig. 4, of the barriers to use of evidence, 'Competing demands/lack of resources and time required to assess the evidence' was the highest ranked choice. Many participants believed their local authority lacked the internal expertise to assess the evidence (rank 2), which was compounded by the inaccessibility of the evidence itself (rank 3). The sense of confusion and uncertainty over the evidence was added to by the perception that there is a lack of evidence relevant to their locality (rank 4).

#### Question 4: the most significant facilitators of local authorities' use of external evidence to inform health/health inequality-related components of climate action plans

**Fig. 5:**
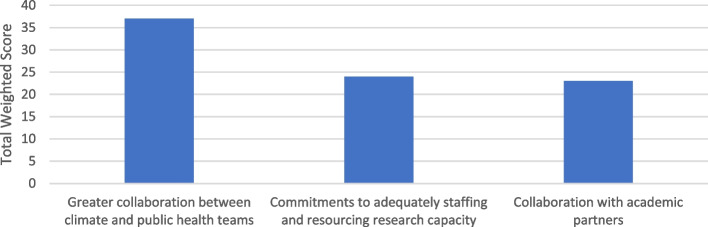
The most significant facilitators of local authorities' use of external evidence to inform health/health inequality-related components of climate action plans

Figure 5 shows the facilitators for use of external evidence. The option ranked in first position by participants was 'greater collaboration between climate and public health teams'. Receiving almost the same overall score, the options in second and third position were 'commitments to adequately staffing and resourcing research capacity' and 'collaboration with academic partners'.

#### Question 5: the most significant barriers to local authorities working with local communities and stakeholders in the process of creating health/health inequality-related components of climate action plans

**Fig. 6:**
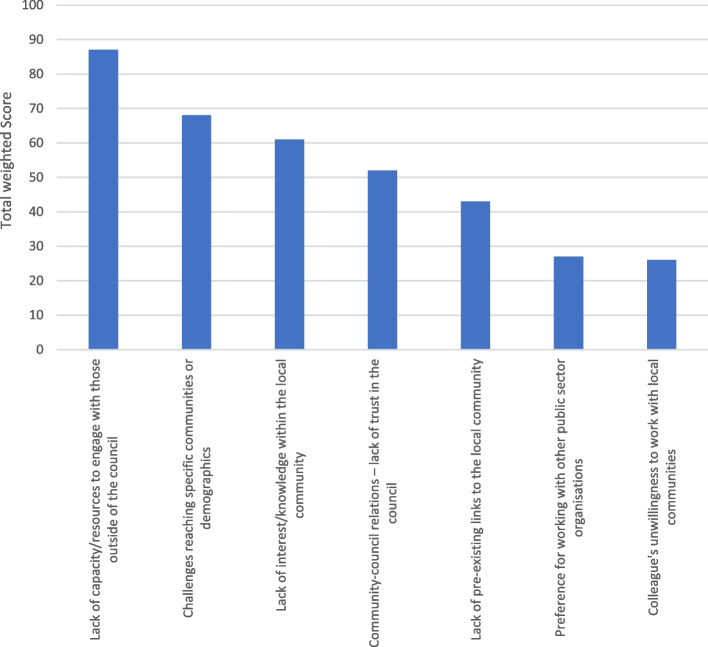
The most significant barriers to local authorities' working with local communities and stakeholders in the process of creating health/health inequality-related components climate action plans

Figure 6 shows the barriers to working with local communities and stakeholders. The first ranked barrier by participants is 'Lack of capacity/resources to engage with those outside of the council'. The second highest ranked barrier was 'challenges reaching specific communities or demographics' highlighting the challenges accessing seldom heard communities. The third-highest barrier of 'lack of interest/knowledge within the local community' may mean many communities are unaware of the value of climate action plans. The next highest ranked barrier 'community-council relations – lack of trust in the council' may point to unfavourable relations with local communities.

#### Question 6: the most significant facilitators of local authorities working with local communities and stakeholders in the process of creating health/health inequality-related components of climate action plans

**Fig. 7:**
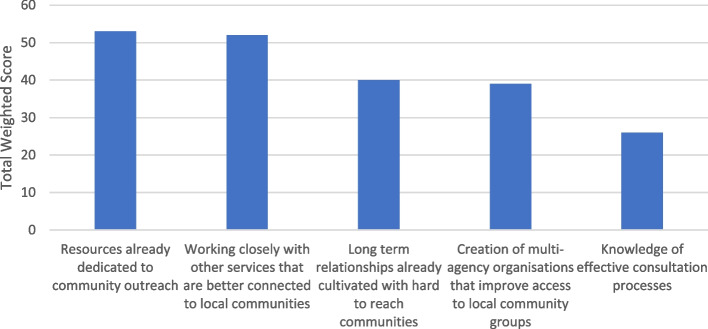
The most significant facilitators of local authorities working with local communities and stakeholders in the process of creating health/health inequality-related components of climate action plans

In Fig. 7, we can see the top ranked facilitators for local authorities working with local communities. The top ranked was resources. The second ranked option was working closely with other services that are better connected to local communities. Third on the list were 'long term relationships already cultivated with hard-to-reach communities'. Fourth ranked was the 'creation of multi-agency organisations that improve access to local community groups'.

#### Question 7: the greatest priorities for local authorities as they identify and tackle the health and health inequality aspects of climate change

**Fig. 8:**
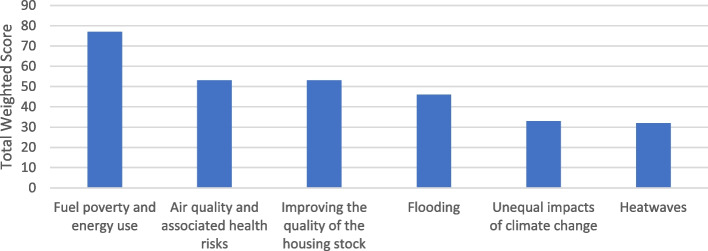
The greatest priorities for local authorities as they identify and tackle the health and health inequality aspects of climate change

Figure 8 presents the ranks of the overall priorities for their local authority as they identify and tackle the health and health inequality aspects of climate change. The top ranked priority for participants was 'fuel poverty and energy use'. Coming in second and third place, with similar rankings, were 'air quality and associated health risks' and 'improving the quality of the housing stock’. The next ranked priorities by participants included 'flooding', 'unequal impacts of climate change', and 'heatwaves', all of which reflect the need to consider the specific vulnerabilities of certain communities and populations when addressing the health effects of climate change. Overall, the data suggests that local authorities prioritise addressing the immediate and tangible effects of climate change on health and well-being, particularly in relation to energy access and the built environment.

### Summary of themes from across the questions

Table 5 show the themes that emerged in ranks 1–4 for each of the six questions from the second-round questionnaire. This table is organised with each column representing one of the six questions and each row corresponding to a barrier or facilitator theme.

**Table 5:** Summary of rankings attributed to each barrier and facilitator in round 2 of the delphi survey

Theme	Q1 – Barriers to implementation of health aspects of climate change plans		Q2—Facilitators of implementation of health aspects of climate change plans	Q3—Barriers to use of evidence to inform the health aspects of climate action plans	Q4—Facilitators of use of evidence to inform the health aspects of climate action plans	Q5—Barriers to working with local communities and stakeholders in creating health aspects of climate action plans		Q6—Facilitators of working with local communities and stakeholders in creating health aspects of climate action plans
Resources, finances, prioritisation	Rank 1—insufficient staff and resources	Rank 1—'the need to save money on energy'		Rank 1—'competing demands/lack of resources and time required to assess the evidence'	Rank 2—'commitments to adequately staffing and resourcing research capacity'	Rank 1—'Lack of capacity/resources to engage with those outside of the council'	Rank 1 – 'Resources already dedicated to community outreach'	
Internal cultural and organisation	Rank 3—'Organisational culture and values/awareness of colleagues (including senior leadership)'	Rank 2—'effective collaboration and joined up working' Rank 3—'the culture and values/awareness of colleagues'					Rank 2—'working closely with other services that are better connected to local communities'	
Policy and political priority	Rank 2- 'The national political and policy context'	Rank 4—"the national political and policy context is conducive"						
Community relations						Rank 2—'challenges reaching specific communities or demographics' Rank 4- 'community-council relations – lack of trust in the council'	Rank 3—'long term relationships already cultivated with seldom heard communities'	
Collaboration with external partners					Rank 1—'greater collaboration between climate and public health teams' Rank 3—In third position was 'collaboration with academic partners'		Rank 4 – 'creation of multi-agency organizations that improve access to local community groups'	
Inaccessibility of external evidence				Rank 2 – Council lacks the internal expertise to assess the evidence Rank 3—inaccessible evidence				

Drawing on the responses from our participants, we identified four recurring themes that appeared most frequently across the questions about various barriers and facilitators. The section below summarises how each of these themes are connected to a range of different barriers and facilitators.

### Finances, resources, personnel, and prioritisation of the issues

Financial and resource constraints were highly ranked as a barrier across a number of domains, including implementation, use of evidence and engaging with communities. Conversely, some respondents ranked finance and resources as a key facilitator for the implementation of health/health inequality-related components of local authorities' climate action plans, use of evidence, and working with local communities.

### Cultural and organisational readiness for the challenge

Barriers related to internal culture and organisation were found to be significant in hindering the implementation of health/health inequality-related components of local authorities' climate action plans. In contrast, for some other respondents, culture and internal practices were more conducive, with internal culture and organisation identified as key facilitators of implementing health/health inequality-related components of local authorities' climate action plans. Regarding facilitators of working with local communities and stakeholders, the second ranked choice was 'working closely with other services that are better connected to local communities'.

### National political and policy context

The national political and policy context is ranked in second position among all barriers to implementation of health/health inequality-related components of climate action plans and thus is perceived as a major hindrance to action by respondents, suggesting some level of unease with wider issues of political will and policy coherence. Ranked fourth of six facilitators of implementation is "the national political and policy context is conducive", suggesting some participants perceived there to be a degree of support from national government and policymakers.

### Collaboration with external partners

To improve access to local community groups, the fourth-ranked facilitator is the 'creation of multi-agency organisations'. This strategy may prove effective in overcoming persistent barriers to co-production, enabling local authorities to work more closely and efficiently with external partners in the pursuit of common goals. In third position among facilitators of use of evidence was 'collaboration with academic partners'. The perception that closer collaboration with academic partners may be beneficial indicates those local authorities with greater access to academic partners, such as those located close to universities or with pre-existing links, may be better positioned to draw on evidence in their climate action plans.

## Discussion

We conducted a two-round survey with people working in local authorities to understand the barriers and facilitators related to considering the health and health inequality impact of climate change in their climate action plans. Our findings showed that personnel and funding required to implement plans is a key barrier, limiting local authorities' ability to draw on external evidence, or engage with their communities. The key facilitators were need to save money on energy, successful partnership working already in place including across local government, with local communities and external stakeholders.

Our results add to a growing literature on local government action for climate change [11–15]. Local government policy on the health impacts of climate change has been little discussed (exceptions include [10, 16]), even though extreme weather and poor air quality are a major concern for the public and seen as a priority for local government [16].

Much of the literature touches on the barriers to effective action by local government, including lack of time and resources, and difficulties in engaging with the wider community [17]. Little in the literature suggests much progress has been made since Porter et al. [15] found that budgetary constraints meant local government could undertake very little in the way of adaptation actions, with the priority on short-term statutory duties. To move climate change back up the ladder of competing priorities, Porter et al. suggest that adaptation can gain traction as an issue when its 'rebranded' as resiliency to extreme weather. What progress there has been, such as the widespread declaration of climate emergencies across local government, has come in response to public pressure to act. Action at the national level is focussed on its statutory obligations legislated for at the national level to reduce carbon emissions to net zero [11]. Another factor that may facilitate action at the local level is the pooling of knowledge between local authorities [18], as may greater public awareness of relevant health exposures in their local area [16].

An informative and balanced snapshot of progress and challenges in UK climate governance is provided by Russell & Christie [24] and their focus on 'multiple levels of county-based government, specifically micro-level action in small towns and parishes.' Their findings show local actors often overcome the lack of national political coordination or backing, with a range of initiatives in operation at different tiers. However, without the minimum standards created by statutory policy, action is inevitably uneven and lacking in evidence to support effectiveness. This results in fragmentary and dislocated multi-level governance, with little opportunity for feedback relationships to form between the various layers of government, and 'no indication of national interest in micro-local climate lessons and experience'. In relation to the financial barriers revealed in our findings, they may be linked to a context in which the functions of local government have been 'hollowed out' [25] due to cuts in funding from central government, leading to a situation in which the 'scope of the state has shrunk locally across England' [25], due to the need to spend remaining funds on core statutory services, including social care for which costs are growing already due to an ageing population. This leaves little to no room for 'discretionary' spending, such as policies to reduce the health risks of climate change. Thus, while national policy documents have acknowledged the need for local authorities to take action [26, 27], there has not been a corresponding allocation of funds, leaving local authorities aware of the challenge they face, but restricting their ability to act. This is a particular issue for local authorities in deprived areas whose budgets have been cut the most [25] and whose communities may be most exposed to health risks from climate change [5].

The latest Local Government Climate Change Survey [28] reveals that 84% of respondents believed to a great extent that lack of funding was a barrier to their authority tackling climate change. This fits in with previous research that noted how funding constraints stymie local progress towards low-carbon policies [29]. As the years pass, the urgency of the situation grows, and this is only compounded by the cost-of-living crisis, as was reflected in the high ranking given in our survey to 'saving money on fuel use' as a facilitator of action. In this context, it is imperative that local authorities receive the necessary support to enact policies that simultaneously reduce the health risks of climate change while lessening the blight of fuel poverty.

The results showed how the culture and organisation of local authorities can either enable or hinder the implementation of policies that protect public health from climate change. Our findings highlight the importance of collaboration and showed that when council colleagues break down the walls between different services and professions they are then well placed to implement policy. On the other hand, when teams work in isolation and fail to share information or collaborate, they will struggle to effectively implement policies and reduce the health impacts of climate change. This finding resonates with previous research that has emphasised the importance of cross-sectoral collaboration for climate action [30].

The diversity of staff teams within local authorities also influences their collaboration with local communities and stakeholders on climate change policy. Our findings showed that councils with a greater breadth of staff teams tend to have more connections to various community groups, facilitating collaboration. This is consistent with the literature that suggests community engagement is vital to 'engage and enthuse the whole of your neighbourhood, in order to reach a shared vision for the future’ and ‘build a consensus around a shared vision for how you would like your area to develop and then design policies to achieve this' [31].

Local authorities that nurture a culture of collaboration and promote democratic decision making are better placed to adapt to the changing climate and deliver effective health-focused interventions. This may mean that, for some councils, addressing the health impacts of climate change requires a culture shift and a reorganisation of internal structures. To the extent that education of officers, and reorganisation of council structures can overcome these problems, local authorities may be better placed to compensate for some of the resource and finance challenges they face.

Action at the local level is undermined by a lack of policy coherence between national level plans and the actions required at a local level to realise them. Most obviously, even though central government has recognised the wide range of emissions over which local government has some influence [26] there remains no statutory requirement for local authorities to enact climate change policies or funding to support them in doing so. This is apparent in our survey results that show problems with securing adequate political support for local climate action, both at the local and national level. All of this combines to weaken the efforts of local authorities to take meaningful steps towards climate resilience and health equity [32].

The localised health risks of climate change present complex and wicked [33] problems that require collaboration across sectors and disciplines to address [34]. Our research showed that collaboration with external partners can be a key facilitator in the creation of effective policies that address the health impacts of climate change. Specifically, our respondents highlighted the importance of multi-agency organisations to improve access to local community groups and collaborating with academic partners to gain access to high-quality research evidence. This underscores the need for local authorities to leverage their access to internal and external expertise to ensure that they can draw on the best available evidence and translate it into effective policy action.

## Strengths and weaknesses

The strength of this study is that we survey a range of people working in local authorities across England to understand the barriers and facilitators to implementing climate change policy that considers the health and health inequality impacts of climate change. We have identified policy levers and tools that local authorities can use to reduce the health and health inequality impact of climate change through both mitigation and adaption. A weakness of the study is the low response rate and small sample size. With a smaller number of participants, the sample may not fully reflect the very diverse range of experiences and perspectives of climate professionals in local government across the whole of England. Caution should therefore be exercised when extrapolating findings to the full range of English local authorities. In addition, although many of the barriers and facilitators discussed could potentially be applicable across the devolved governments of the UK, a limitation of our study is we were only able to recruit one local authority officer from outside England (from Wales)'. However, even though we have a small sample size our sample has diversity across local authority structures: London Borough, Unitary Authority (Blackpool), District (Wyre) County (e.g. Lancashire County Council) and one of the Mayoral Combined Authorities (e.g. Greater Manchester) which enhances the potential applicability of our findings across England.

## Conclusion

Resource constraints, institutional fragmentation, lack of political support, and competing priorities pose significant challenges for local authorities seeking to implement climate action plans that prioritise health equity. To overcome these barriers, local authorities must nurture a culture of innovation, collaboration, and purpose, while also addressing the urgent need to protect vulnerable communities from the health impacts of climate change. This means not just working with external partners, but also collaborating and co-producing with communities to achieve health equity and mitigate the debilitating effect of climate change on public health. Failure to do so risks exacerbating existing health inequities and compromising the long-term sustainability of local communities. Lastly, the overriding and unavoidable conclusion is that local authorities cannot solve these problems on their own—policymakers must provide the resources and support that local authorities need to make a real difference. Because of their unique position and roles within local areas, local government can play a key role in facilitating adaptive and mitigating measures to reduce the health and health inequality impacts of climate change and maximise the co-benefits of these measures.

### Supplementary Information

**Additional file 1: Appendix 1.** First Round Survey. **Appendix 2.** Second Round Survey. **Appendix 3.** Analysis of data from second round of survey.

## Data Availability

The datasets used and/or analysed during the current study are available from the corresponding author on reasonable request.
