# Supplementary material for: Assessing the barriers and facilitators of climate action planning in local governments: a two-round survey of expert opinion
Source: BMC Public Health. 2023 Oct 5;23:1932. doi: 10.1186/s12889-023-16853-8 (PMC10557327; doi:10.1186/s12889-023-16853-8)

# Appendix 1. First Round Survey

Link to first round survey: [Qualtrics Survey | Qualtrics Experience Management](https://lancasteruni.eu.qualtrics.com/jfe/form/SV_5u8fp7Ex3CHpBxY)

# Climate change/health survey - round 1

**Start of Block: Background information and Consent**

| 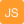 |
| --- |

PIS
**Participant Information Sheet

 Exploring the evidence gaps related to the actions and priorities of local authorities for mitigating the health and health inequality impacts of climate change.**
 We are a team of researchers based in the Division of Health Research. We are conducting this research on behalf of Lancaster University, in collaboration with Lancaster Environment Centre and Blackpool Council.

 **What is the study about?**
 The purpose of this study is to identify the actions and priorities of local authorities for mitigating the health and health inequality impacts of climate change.

 **Why have I been approached?**
 You have been approached because the study requires information from key stakeholders within local authorities who have set net zero climate action targets for 2030 and/or 2050. We are looking for a representative sample of climate change and/or sustainability leads who have knowledge and expertise within this sector, so your insights and expertise would be invaluable. Please note, you do not have to be specifically located within the public health department within the council.

 **Do I have to take part?**
 No. It’s completely up to you to decide whether or not you take part. If you decide to take part, please keep a copy of this information sheet. You will also be asked to sign a consent form.

 **What will I be asked to do if I take part?**
 You will be asked to fill out a questionnaire about your local authority’s climate action plan and the extent to which it considers actions for mitigating the health and health inequality impacts of climate change.
 You will also be invited to fill out a second questionnaire at a later time. The group results from the first questionnaire will be presented, and you will be asked to rank the broad group responses according to importance.

 **Will my data be identifiable?**
 All data collected for this study will be made anonymous through the use of pseudonyms and stored securely:
   Any personal information (including name and contact details) will be confidential (no-one other than the research team will be able to access it). In line with the Data Protection Act 2018, it will be kept on an encrypted and password protected hard drive and kept separately from your responses to the questionnaires. All personal information will be deleted on completion of the project. The online questionnaire files will be encrypted (meaning that no-one other than the researcher will be able to access them) and stored on a University-approved secure and password-protected hard drive.
 At the end of the project’s timeframe, all research data will be kept securely and accessible, according to Lancaster University’s Research Data Management policy (a minimum of 10 years) and will be made accessible to other academic researchers, according to NIHR’s position on sharing of research data. This will be done to encourage the accessibility of research data and for other researchers to review or re-analyse data from publicly funded research.

 Under the GDPR, participants have certain rights when personal data is collected about them. They have the right to access any personal data held about them, to object to the processing of their personal information, to rectify personal data if it is inaccurate, the right to have data about them erased and, depending on the circumstances, the right to data portability. Please be aware that many of these rights are not absolute and only apply in certain circumstances. If you would like to know more about participants’ rights in relation to their personal data, please speak to the researchers.

 **What will happen to the results?**
 The main outputs of this research will be a description of key research questions and priorities to inform the future funding call for NIHR. This funding call aims to progress the climate change agenda within a Local Authority context with a health and health inequality lens. We will also produce a report outlining the methodology and key findings as well as peer-reviewed research outputs.

 **Are there any risks?**
 There are no risks anticipated with participating in this study. However, if you experience any distress following participation you are encouraged to inform the researchers and contact the resources provided at the end of this sheet.

 **Are there any benefits to taking part?**
 Although you may find participating interesting, there are no direct benefits in taking part. The wider benefits of this research aim to identify and address climate change actions and priorities with a health and health inequality focus which may have a public health benefit.

 **Who has reviewed the project?**
 This study has been reviewed and approved by the Faculty of Health and Medicine Research Ethics Committee at Lancaster University.

 **Where can I obtain further information about the study if I need it?**
 if you have any questions about the study, please contact the research team:
**Dr Steven Dodd**, Researcher, Division of Health Research, Lancaster University, email: s.r.dodd2@lancaster.ac.uk
 **Prof Heather Brown,** Co-Principal Investigator, Division of Health Research, Health Innovation One, Lancaster University, Sir John Fischer Drive, Lancaster, LA1 4AT, email: h.w.brown@lancaster.ac.uk
  
**Complaints**
 
If you wish to make a complaint or raise concerns about any aspect of this study and do not want to speak to the researcher, you can contact:

 **Dr Laura Machin**
 Email: l.machin@lancaster.ac.uk
 Chair, Faculty of Health and Medicine Research Ethics Committee
 Lancaster Medical School
 Lancaster University
 LA1 4AT

 Thank you for taking the time to read this information sheet.

 **Resources in the event of distress**
 **I**n the unlikely event that you should you feel distressed either as a result of taking part, or in the future, the following resources may be of assistance. 
 ​​​​​​​
 [NHS Mental Health Services](https://www.nhs.uk/nhs-services/mental-health-services/)

| 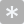 |
| --- |

Consent Form
**Consent Form
 Study Title: Exploring the evidence gaps related to the actions and priorities of Local Authorities for mitigating the health and health inequality impacts of climate change**
 We are asking if you would like to take part in a research project which aims to identify the actions and priorities of local authorities for mitigating the health and health inequality impacts of climate change. Before you consent to participating in the study, we ask that you read the participant information sheet and tick each box below if you agree. If you have any questions or queries before signing the consent form, please speak to the Co-Principal Investigator, Prof. Heather Brown.

- I confirm that I have read and understood the information sheet and fully understand what is expected of me within this study. (1)
- I confirm that I have had the opportunity to consider the information, ask questions and have had these answered satisfactorily. (4)
- I understand that my participation is voluntary and that I am free to withdraw from the study at any time without giving any reason. (5)
- I understand that once my questionnaire data has been anonymised and incorporated into the analysis, it might not be possible for it to be withdrawn, though every attempt will be made to extract my data, up to the point of publication. (6)
- I understand that the information from my questionnaire responses will be pooled with other participants’ responses, anonymised and may be published; all reasonable steps will be taken to protect the anonymity of the participants involved in this study. (7)
- I consent to information and quotations from my questionnaire being used in reports, conferences, publications and training events. (8)
- I understand that any personal information I give will remain confidential and all information anonymous unless it is thought that there is a risk of harm to myself or others, in which case the co-principal investigator may need to share this information with a relevant authority. (9)
- I consent to Lancaster University archiving questionnaire responses for at least 10 years after the study has finished as part of their Research Data Management Policy, and that the anonymised data can be made available to other academic researchers to review and/or re-analyse. (10)
- I consent to take part in the above study. (11)

Employment details Please provide type your signature and the date on which you begin completing the survey

- Signature (1) __________________________________________________
- Date (2) __________________________________________________

Q32 Please provide type your signature and the date on which you begin completing the survey

- Signature (1) __________________________________________________
- Date (2) __________________________________________________

Contact details We would like to invite you to take part in a second questionnaire, for which we need your contact details. Please provide us with your email address if you are happy for us to contact you to take part in a second questionnaire:

________________________________________________________________

**End of Block: Background information and Consent**

**Start of Block: Introduction and collection of employment information**

Introduction The questionnaire below forms part of a research project about the actions and priorities of local authorities for mitigating the health and health inequality impacts of climate change. As part of this research, we would like to engage with local authority officers who can provide vital insights on this matter.

The intended outcome of this study will be to generate a list of barriers, facilitators and potential priorities and solutions to tackling the health and health inequality impacts of climate change within local authorities. Project outputs will be used to inform the National Institute for Health and Care Research's future funding call concerning the health and health inequality aspects of local authorities climate change policies.

There are 25 questions and the survey should take approximately 25 minutes to complete.

The study is undertaken for the National Institute of Health Research (NIHR) Public Health Research Programme. If you have any questions, please do not hesitate to contact us: Prof Heather Brown, Division of Health Research, Lancaster University, h.w.brown@lancaster.ac.uk.

Please begin the survey by entering some basic information about yourself below:

- Job role (1) __________________________________________________
- Number of years in role (2) __________________________________________________
- Years of experience in local government (3) __________________________________________________

**End of Block: Introduction and collection of employment information**

**Start of Block: Development of Climate Action Plan**

Q1/25 Q1/25: Please indicate the extent to which you believe Climate Action Plans should consider risks to health and health inequalities

|  | To a Great Extent (1) | Somewhat (2) | Very little (3) | Not at all (4) | Unsure (5) |
| --- | --- | --- | --- | --- | --- |
| Select one of five options (1) |  |  |  |  |  |

Q2/25 Q2/25: Please indicate the extent to which consideration of health and health inequalities form a part of your council's Climate Action Plan

|  | To a Great Extent (1) | Somewhat (2) | Very little (3) | Not at all (4) | Unsure (5) |
| --- | --- | --- | --- | --- | --- |
| Select one of five options (1) |  |  |  |  |  |

*Skip To: Q10/25 If Q2/25: Please indicate the extent to which consideration of health and health inequalities form a... = Select one of five options [ Not at all ]*

Q3/25 Q3/25: Please indicate the extent to which risks to the health of specific communities or demographic groups form part of your Climate Action Plan

|  | To a Great Extent (1) | Somewhat (2) | Very little (3) | Not at all (4) | Unsure (5) |
| --- | --- | --- | --- | --- | --- |
| Select one of five options (1) |  |  |  |  |  |

Q4/25 Q4/25: Please indicate the extent to which your council's Climate Action Plan has drawn on externally produced evidence to inform strategies to identify and mitigate health-related impacts of climate change

|  | To a Great Extent (1) | Somewhat (2) | Very little (3) | Not at all (4) | Unsure (5) |
| --- | --- | --- | --- | --- | --- |
| Select one of five options (1) |  |  |  |  |  |

Q5/25 Q5/25: Does your council's Climate Action Plan make reference to any indirect impacts (positive or negative) on health and health inequalities due to specific mitigation/adaption actions included in the Plan?

- Yes (1)
- No (2)

*Display This Question:*

*If Q5/25: Does your council's Climate Action Plan make reference to any indirect impacts (positive o... = Yes*

Q6/25 Q6/25: Please summarise the risks to public health included within the Climate Action Plan.

________________________________________________________________

________________________________________________________________

________________________________________________________________

________________________________________________________________

________________________________________________________________

*Display This Question:*

*If Q5/25: Does your council's Climate Action Plan make reference to any indirect impacts (positive o... = No*

Q7/25 Q7/25: Please summarise why, at this stage, risks to health and public health do not form part of the council's Climate Action Plan

________________________________________________________________

________________________________________________________________

________________________________________________________________

________________________________________________________________

________________________________________________________________

Q8/25 Q8/25: Please summarise the forms and sources of evidence drawn upon for the health-related components of your Climate Action Plan

________________________________________________________________

________________________________________________________________

________________________________________________________________

________________________________________________________________

________________________________________________________________

Q9/25 Q9/25: Please indicate the extent to which your council has continued to gather evidence to support implementation and evaluation of the health-related components of its Climate Action Plan.

|  | To a Great Extent (1) | Somewhat (2) | Very little (3) | Not at all (4) | Unsure (5) |
| --- | --- | --- | --- | --- | --- |
| Select one of five options (1) |  |  |  |  |  |

Q10/25 Q10/25: Please summarise any barriers you believe may have limited your council's use of externally produced evidence in the formulation and implementation of the health-related components of its Climate Action Plan

________________________________________________________________

________________________________________________________________

________________________________________________________________

________________________________________________________________

________________________________________________________________

**End of Block: Development of Climate Action Plan**

**Start of Block: Co-production of the health-related aspects of your Climate Action Plan**

Q11/25 Q11/25: Please indicate the extent to which you believe the health-related components of Climate Action Plans should be co-produced with local communities and organisations?

|  | To a Great Extent (1) | Somewhat (2) | Very little (3) | Not at all (4) | Unsure (5) |
| --- | --- | --- | --- | --- | --- |
| Select one of five options (1) |  |  |  |  |  |

Q12/25 Q12/25: Please indicate the extent to which the health-related components of your Climate Action Plan are the product of consultation or collaboration with local communities and organisations?

|  | To a Great Extent (1) | Somewhat (2) | Very little (3) | Not at all (4) | Unsure (5) |
| --- | --- | --- | --- | --- | --- |
| Select one of five options (1) |  |  |  |  |  |

Q13/25 Q13/25: Please indicate the extent to which the health-related components of your Climate Action Plan are a product of engaging with hard-to-reach communities and under-represented groups?

|  | To a Great Extent (1) | Somewhat (2) | Very little (3) | Not at all (4) | Unsure (5) |
| --- | --- | --- | --- | --- | --- |
| Select one of five options (1) |  |  |  |  |  |

Q14/25 Q14/25: Please indicate the extent to which your Climate Action Plan involved establishing partnerships with, and securing commitments from, different stakeholders and organisations within your community?

|  | To a Great Extent (1) | Somewhat (2) | Very little (3) | Not at all (4) | Unsure (5) |
| --- | --- | --- | --- | --- | --- |
| Select one of five options (1) |  |  |  |  |  |

Q15/25 Q15/25: Please provide details of how your council worked with the local community when developing the health-related components of your Climate Action Plan, including which communities and stakeholders were involved and how.

________________________________________________________________

________________________________________________________________

________________________________________________________________

________________________________________________________________

________________________________________________________________

Q16/25 Q16/25: Please describe what you think are the greatest benefits of working with local communities and stakeholders when developing the health-related components of a Climate Action Plan

________________________________________________________________

________________________________________________________________

________________________________________________________________

________________________________________________________________

________________________________________________________________

Q17/25 Q17/25: Please describe what you think are the greatest barriers to working with local communities when developing the health-related components of a Climate Action Plan

________________________________________________________________

________________________________________________________________

________________________________________________________________

________________________________________________________________

________________________________________________________________

**End of Block: Co-production of the health-related aspects of your Climate Action Plan**

**Start of Block: Implementation of health-related aspects of your Climate Action Plan**

Q18/25 Q18/25: Please indicate the extent to which you believe your council has made good progress towards implementing the health-related components of its Climate Action Plan

|  | To a Great Extent (1) | Somewhat (2) | Very little (3) | Not at all (4) | Unsure (5) |
| --- | --- | --- | --- | --- | --- |
| Select one of five options (1) |  |  |  |  |  |

Q19/25 Q19/25: Please indicate the extent to which you believe the health-related components of your Climate Action Plan are a priority for your council as a whole

|  | To a Great Extent (1) | Somewhat (2) | Very little (3) | Not at all (4) | Unsure (5) |
| --- | --- | --- | --- | --- | --- |
| Select one of five options (1) |  |  |  |  |  |

Q20/25 Q20/25: Please indicate the extent to which working practices within your council have been affected by adoption of the health-related components of your Climate Action Plan.

|  | To a Great Extent (1) | Somewhat (2) | Very little (3) | Not at all (4) | Unsure (5) |
| --- | --- | --- | --- | --- | --- |
| Select one of five options (1) |  |  |  |  |  |

Q21/25 Q21/25: Please summarise how progress towards the health-related components of your Climate Action Plan will be assessed

________________________________________________________________

________________________________________________________________

________________________________________________________________

________________________________________________________________

________________________________________________________________

Q22/25 Q22/25: Please summarise the barriers to implementing the health-related components of your council's Climate Action Plan

________________________________________________________________

________________________________________________________________

________________________________________________________________

________________________________________________________________

________________________________________________________________

Q23/25 Q23/25: Do you think having effective policies for addressing the health-related impacts of climate change is compatible with current ways of working (e.g. culture) in your council/local authority?

- Yes (1)
- No (2)

*Display This Question:*

*If Q23/25: Do you think having effective policies for addressing the health-related impacts of clima... = Yes*

Q24/25 Q24/25: What is it about your council that has made implementing an effective Climate Action Plan compatible with current ways of working?

________________________________________________________________

________________________________________________________________

________________________________________________________________

________________________________________________________________

________________________________________________________________

*Display This Question:*

*If Q23/25: Do you think having effective policies for addressing the health-related impacts of clima... = No*

Q25/25 Q25/25: Why do you feel that implementing an effective Climate Action Plan is incompatible with current ways of working in your council/local authority?

________________________________________________________________

________________________________________________________________

________________________________________________________________

________________________________________________________________

________________________________________________________________

**End of Block: Implementation of health-related aspects of your Climate Action Plan**

# Appendix 2. Second Round Survey

Link to second round survey: [Qualtrics Survey | Qualtrics Experience Management](https://lancasteruni.eu.qualtrics.com/jfe/form/SV_efmzY8AseQeiSFw)

# Climate change/health survey - round 2

**Start of Block: Block 1**

Q7 Exploring the evidence gaps related to the actions and priorities of local authorities for mitigating the health and health inequality impacts of climate change.
 Delphi Survey - Round Two

 The second survey uses data from the first survey from you and other participants to generate a list of barriers and facilitators related to the creation and implementation of the health/health inequality aspects of your council’s Climate Action Plan. In this survey we want you to rank these barriers and facilitators in order of significance.   

 **Participant Information Sheet**

 Who has reviewed the project?
 This study has been reviewed and approved by the Faculty of Health and Medicine Research Ethics Committee at Lancaster University.

 Where can I obtain further information about the study if I need it?
 if you have any questions about the study, please contact the research team:
 Dr Steven Dodd, Researcher, Division of Health Research, Lancaster University, email: s.r.dodd2@lancaster.ac.uk
 Prof Heather Brown, Co-Principal Investigator, Division of Health Research, Health Innovation One, Lancaster University, Sir John Fischer Drive, Lancaster, LA1 4AT, email: h.w.brown@lancaster.ac.uk
  
 Complaints
  
 If you wish to make a complaint or raise concerns about any aspect of this study and do not want to speak to the researcher, you can contact:

 Dr Laura Machin
 Email: l.machin@lancaster.ac.uk
 Chair, Faculty of Health and Medicine Research Ethics Committee
 Lancaster Medical School
 Lancaster University
 LA1 4AT

 Thank you for taking the time to read this information sheet.

 Resources in the event of distress
 In the unlikely event that you should you feel distressed either as a result of taking part, or in the future, the following resources may be of assistance. 

 NHS Mental Health Services

Q10 Please provide type your signature, email address and the date on which you begin completing the survey. Your email address will help us to connect the results of your second survey to your first.

- Signature (1) __________________________________________________
- Date (2) __________________________________________________
- Email address (3) __________________________________________________

**End of Block: Block 1**

**Start of Block: Default Question Block**

Q1/7 Please 'drag and drop' the options below to rank them according to which you consider to be the most significant **barriers** to implementation of the health/health inequality-related components of your Climate Action Plan (1 = most significant, 11 = least significant)

______ Insufficient staff and resources (1)

______ Organisational culture, values/awareness of colleagues (including senior leadership) (2)

______ Council colleagues working in silos, lack of joining up and collaboration (3)

______ The difficulty of understanding the health effects of climate change (4)

______ National political and policy context not conducive (5)

______ Local political context not conducive (6)

______ Difficulties engaging with colleagues from the healthcare system (7)

______ Difficulties engaging with local communities and stakeholders (8)

______ Priority is narrowly on reducing emissions and not on health (9)

______ Too little is known about the health impacts of climate change (10)

______ Health not thought to be at risk from climate change in your locality (11)

Q2 Please rank the options below according to which you consider to be the most significant **facilitators** of the implementation of the health/health inequality-related components of your Climate Action Plan (1 = most significant, 6 = least significant)

______ The culture and values/awareness of colleagues (including senior leadership) (1)

______ The national political and policy context is conducive (2)

______ Effective collaboration and joined up working (3)

______ Pre-existing commitment to working with all sections of the local community (4)

______ The need to save money on energy expenditure is conducive to addressing the issues (5)

______ The shift to working from home is conducive to addressing the issues (6)

Q3 Please rank the options below according to which you consider to be the most significant barriers to your local authority's use of external evidence to inform the health/health inequality-related components of your Climate Action Plan (1 = most significant, 6 = least significant)

______ Competing demands/lack of resources and time required to assess the evidence (1)

______ Inaccessibility of the evidence (2)

______ Lack of evidence at a local level (3)

______ Lack of expertise required to assess the evidence (4)

______ Lack of commitment on the part of senior leadership (5)

______ Lack of political support at a local level (6)

Q4 Please rank the options below according to which you consider to be the most significant **facilitators** of your local authority's use of external evidence to inform the health/health inequality-related components of your Climate Action Plan (1 = most significant, 3 = least significant)

______ Collaboration with academic partners (1)

______ Commitments to adequately staffing and resourcing research capacity (2)

______ Greater collaboration between climate and public health teams (3)

Q5 Please rank the options below according to which you consider to be the most significant **barriers** to your local authority working with local communities and stakeholders in the process of creating the health/health inequality-related components of your Climate Action Plan (1 = most significant, 7 = least significant)

______ Lack of capacity/resources to engage with those outside of the council (1)

______ Challenges reaching specific communities or demographics (2)

______ Community-council relations – lack of trust in the council (3)

______ Lack of interest/knowledge within the local community (4)

______ Lack of pre-existing links to the local community (5)

______ Colleague's unwillingness to work with local communities (6)

______ Preference for working with other public sector organisations (7)

Q6 Please rank the options below according to which you consider to be the most significant facilitators of your local authority working with local communities and stakeholders in the process of creating the health/health inequality-related components of your Climate Action Plan (1 = most significant, 5 = least significant) - Resources already dedicated to community outreach

______ Resources already dedicated to community outreach (1)

______ Long term relationships already cultivated with hard to reach communities (2)

______ Working closely with other services that are better connected to local communities (3)

______ Creation of multi-agency organisations that improve access to local community groups (4)

______ Knowledge of effective consultation processes (5)

Q7 Please rank the options below according to which you consider to be the greatest priority for your local authority as it identifies and tackles the health and health inequality aspects of climate change (1 = most significant, 6 = least significant)

______ Flooding (1)

______ Air quality and associated health risks (2)

______ Heatwaves (3)

______ Improving the quality of the housing stock (4)

______ Fuel poverty and energy use (5)

______ Unequal impacts of climate change (6)

**End of Block: Default Question Block**

# Appendix 3. Analysis of data from second round of survey

Question 1. The most significant barriers to implementation of health/health inequality-related components of local authorities' climate action plans

|  | **Total** | **Rank** |
| --- | --- | --- |
| **Insufficient staff and resources** | **134** | **1** |
| **National political and policy context not conducive** | **107** | **2** |
| **Organisational culture, values/awareness of colleagues (including senior leadership)** | **104** | **3** |
| **Council colleagues working in silos, lack of joining up and collaboration** | **89** | **4** |
| **Priority is narrowly on reducing emissions and not on health** | **88** | **5** |
| **Difficulties engaging with local communities and stakeholders** | **77** | **6** |
| **Difficulties engaging with colleagues from the healthcare system** | **75** | **7** |
| **The difficulty of understanding the health effects of climate change** | **69** | **8** |
| **Local political context not conducive** | **66** | **9** |
| **Health not thought to be at risk from climate change in your locality** | **59** | **10** |
| **Too little is known about the health impacts of climate change** | **56** | **11** |

Question 2: The most significant facilitators of the implementation of health/health inequality-related components of local authorities' climate action plans

|  | **Total** | **Rank** |
| --- | --- | --- |
| **The need to save money on energy expenditure is conducive to addressing the issues** | **59** | **1** |
| **Effective collaboration and joined up working** | **58** | **2** |
| **The culture and values/awareness of colleagues (including senior leadership)** | **56** | **3** |
| **The national political and policy context is conducive** | **53** | **4** |
| **Pre-existing commitment to working with all sections of the local community** | **41** | **5** |
| **The shift to working from home is conducive to addressing the issues** | **27** | **6** |

Question 3: The most significant barriers to local authorities' use of external evidence to inform the health/health inequality-related components of their climate action plans

|  | Total | Rank |
| --- | --- | --- |
| **Competing demands/lack of resources and time required to assess the evidence** | 76 | 1 |
| **Lack of expertise required to assess the evidence** | 54 | 2 |
| **Inaccessibility of the evidence** | 50 | 3 |
| **Lack of evidence at a local level** | 48 | 4 |
| **Lack of commitment on the part of senior leadership** | 39 | 5 |
| **Lack of political support at a local level** | 27 | 6 |
|  |  |  |

Question 4: The most significant facilitators of local authorities' use of external evidence to inform health/health inequality-related components of their climate action plans:

|  | **Total** | **Rank** |
| --- | --- | --- |
| **Greater collaboration between climate and public health teams** | **37** | **1** |
| **Commitments to adequately staffing and resourcing research capacity** | **24** | **2** |
| **Collaboration with academic partners** | **23** | **3** |

Question 5: The most significant barriers to local authorities working with local communities and stakeholders in the process of creating health/health inequality-related components of climate action plans

|  | **Total** | **Rank** |
| --- | --- | --- |
| **Lack of capacity/resources to engage with those outside of the council** | **87** | **1** |
| **Challenges reaching specific communities or demographics** | **68** | **2** |
| **Lack of interest/knowledge within the local community** | **61** | **3** |
| **Community-council relations – lack of trust in the council** | **52** | **4** |
| **Lack of pre-existing links to the local community** | **43** | **5** |
| **`Preference for working with other public sector organisations** | **27** | **6** |
| **Colleague's unwillingness to work with local communities** | **26** | **7** |

Question 6: The most significant facilitators of local authorities working with local communities and stakeholders in the process of creating health/health inequality-related components of climate action plans:

|  | **Total** | **Rank** |
| --- | --- | --- |
| **Resources already dedicated to community outreach** | **53** | **1** |
| **Working closely with other services that are better connected to local communities** | **52** | **2** |
| **Long term relationships already cultivated with hard to reach communities** | **40** | **3** |
| **Creation of multi-agency organisations that improve access to local community groups** | **39** | **4** |
| **Knowledge of effective consultation processes** | **26** | **5** |

Question 7: The greatest priorities for local authorities as they identify and tackle the health and health inequality aspects of climate change

|  | **Total** | **Rank** |
| --- | --- | --- |
| **Fuel poverty and energy use** | **77** | **1** |
| **Air quality and associated health risks** | **53** | **2** |
| **Improving the quality of the housing stock** | **53** | **2** |
| **Flooding** | **46** | **4** |
| **Unequal impacts of climate change** | **33** | **5** |
| **Heatwaves** | **32** | **6** |
|  |  |  |


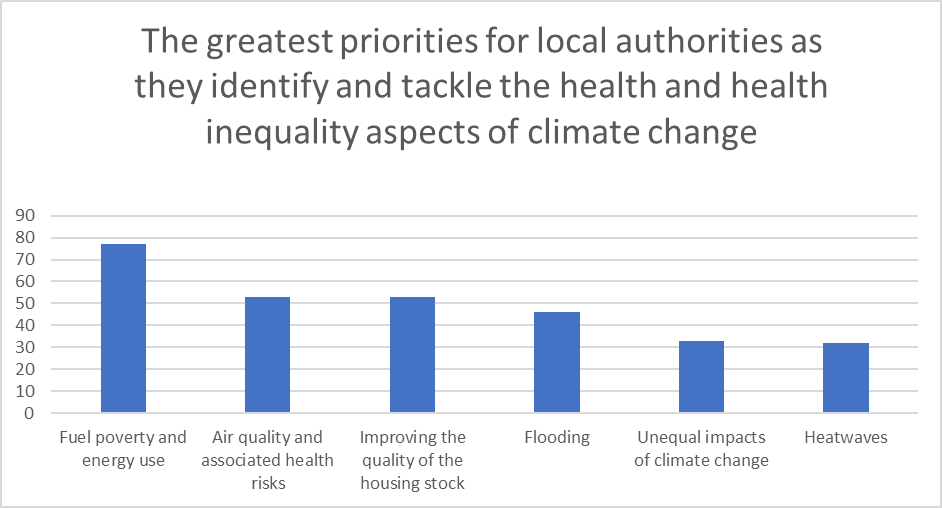

Supplement: Supplementary file 1 — Additional file 1: Appendix 1. First Round Survey. Appendix 2. Second Round Survey. Appendix 3. Analysis of data from second round of survey. [file 12889_2023_16853_MOESM1_ESM.docx]
